# Plasma-Lyte-148 Versus Normal Saline 0.9% in Diabetic Ketoacidosis Management: A Review

**DOI:** 10.7759/cureus.41079

**Published:** 2023-06-28

**Authors:** Mutaz I Othman, Abdulqadir J Nashwan, Moayad Alfayoumi, Mohamad Khatib, Ahmad A Abujaber

**Affiliations:** 1 Critical Care, Hamad Medical Corporation, Doha, QAT; 2 Pharmacy, Hamad Medical Corporation, Doha, QAT; 3 Nursing, Hamad Medical Corporation, Doha, QAT

**Keywords:** renal replacement therapy, fluid resuscitation, diabetic ketoacidosis, normal saline 0.9%, plasma-lyte-148

## Abstract

Diabetic ketoacidosis (DKA) is a critical complication of diabetes mellitus characterized by hyperglycemia, ketonemia, circulatory collapse, hypokalemia, and metabolic acidosis. The therapeutic management of DKA includes vigilant fluid resuscitation to address dehydration and electrolyte imbalances and restore hemodynamic stability. The choice of fluid, either isotonic saline or a balanced electrolyte solution like Plasma-Lyte 148 (PL), is pivotal in the clinical outcomes of DKA patients. Recent studies have compared the effectiveness of these fluid solutions in DKA management, focusing on different clinical outcomes such as the resolution of metabolic acidosis, electrolyte imbalances, the incidence of acute kidney injury, and length of hospital stay. This review examines the literature comparing isotonic saline and balanced electrolyte solutions for fluid resuscitation in DKA, analyzing the associated clinical outcomes. Through synthesizing research findings, this review aims to elucidate the efficacy and potential advantages of utilizing PL as an alternative to traditional isotonic saline for fluid resuscitation in treating DKA. This would further facilitate evidence-based decision-making among healthcare professionals and contribute to optimizing DKA management strategies. Understanding the intricacies and implications of fluid resuscitation is crucial, given its profound impact on patient outcomes in DKA management.

## Introduction and background

Fluid resuscitation is crucial in managing Diabetic Ketoacidosis (DKA), a life-threatening complication of diabetes characterized by hyperglycemia, ketosis, and metabolic acidosis [1]. In DKA, insulin deficiency leads to an accelerated breakdown of fatty acids, producing ketone bodies that contribute to acidosis [1]. Concurrently, hyperglycemia induces osmotic diuresis, resulting in significant fluid and electrolyte losses. Fluid resuscitation aims to reverse these detrimental effects [1]. Replenishing intravascular volume mitigates hypovolemia, improving tissue perfusion and oxygen delivery. It also dilutes the concentration of glucose and ketones in the blood, helping to correct hyperglycemia and acidosis [1].

Furthermore, restoring renal blood flow facilitates the excretion of glucose and ketones through the urine, which is vital for clearing ketone bodies and preventing the progression of metabolic acidosis [2]. Additionally, fluid replacement helps reestablish electrolyte balance, particularly for potassium which can be dangerously depleted in DKA [2]. Timely and appropriate fluid resuscitation is indispensable in stabilizing a patient's hemodynamics and averting the potentially fatal consequences of DKA [2]. Healthcare professionals must exercise vigilance and precision in selecting the types and rates of fluids administered to optimize patient outcomes. Intravenous fluids are crucial in replenishing depleted fluid volumes across intravascular, interstitial, and intracellular spaces. Initially, isotonic saline is favored to reestablish circulation and mitigate the risks associated with cerebral edema [3-4]. 

A holistic approach to managing DKA involves administering fluids, insulin, and electrolyte replacement concurrently. The overarching goal is to restore normal metabolic and physiological functions, thereby relieving ketoacidosis and hyperglycemia. Healthcare professionals involved in treating DKA must possess an in-depth understanding of fluid resuscitation principles and challenges due to its vital role in patient outcomes [5]. Plasma-Lyte 148 (PL) is a balanced, sterile intravenous fluid solution frequently used for patient rehydration and electrolyte restoration. This solution contains an amalgamation of electrolytes, including sodium, chloride, potassium, magnesium, and calcium. The term "148" signifies the sodium concentration of 148 milliequivalents per liter present in the solution. PL is often deployed to achieve fluid and electrolyte balance in patients who require intravenous rehydration, particularly in cases of dehydration, electrolyte disturbances, or surgical procedures [6-7]. This review evaluates and contrasts the effects of isotonic saline and hypotonic solutions in addressing fluid and electrolyte deficiencies in DKA patients. Moreover, it aims to identify best practices for fluid management in DKA treatment, focusing on factors such as fluid type, administration rate, and monitoring protocols. Additionally, the review investigates the effects of fluid resuscitation on clinical outcomes such as hospital stay duration, mortality rates, and resolution of ketoacidosis in patients with DKA.

## Review

This review explores various research studies centered around managing Diabetic Ketoacidosis (DKA), particularly focusing on fluid resuscitation methods. The comparison between Plasma-Lyte (PL) and Normal Saline (NS) in terms of efficacy in treating DKA forms the cornerstone of multiple analyses, with investigations spanning different patient demographics and incorporating various outcome measures. Notably, these studies dive into the realms of metabolic acidosis, acute kidney injury (AKI), and the potential of certain biomarkers for early detection of AKI in pediatric patients with DKA. The review further encapsulates research on fluid administration modes and their impact on DKA resolution and a unique case study linking severe hypercalcemia with DKA. The culminating focus is preventing hyperchloremic metabolic acidosis in DKA patients, illustrating the complexity and multifaceted nature of managing this serious diabetes complication.

In a Phase 2 trial involving 90 adults with severe DKA in ICUs, PL was compared to NS 0.9% (SC) to evaluate their efficacy in resolving DKA. At 24 hours, 69% of patients treated with PL achieved DKA resolution, compared to 36% in the SC group. However, by 48 hours, the difference was not statistically significant (96% in PL versus 86% in SC). The study also found that PL led to faster resolution of metabolic acidosis without increasing ketosis. Lengths of ICU and hospital stays were slightly shorter for the PL group. The authors recommend confirmation through a larger Phase 3 trial [7].

Chua et al. conducted a multi-center retrospective analysis comparing the efficacy of Plasma-Lyte (PL) and Normal Saline (NS) in fluid resuscitation for DKA [8]. The study revealed that PL more rapidly ameliorated metabolic acidosis than NS, and patients treated with PL showed greater median improvements in serum bicarbonate and base excess levels. PL electrolytes-sodium, potassium, magnesium, and chloride-ensure proper cellular functioning and acid-base balance, and its unique components, acetate and gluconate, metabolize into bicarbonate, neutralizing blood acidity, aiding in metabolic acidosis correction and transiently improving blood pressure. However, no significant difference was observed in glycemic regulation or the duration of intensive care unit (ICU) stays between the two groups.

From a different perspective, Attokaran et al. [9] investigated whether intravenous (IV) insulin administration via a peripheral line was more effective than subcutaneous (SC) administration in resolving DKA without exacerbating ketosis. In this randomized phase 2 trial involving 90 adult ICU patients, results indicated that 69% of PL patients resolved DKA within 24 hours compared to 36% of SC patients. Furthermore, PL patients experienced shorter ICU and hospital stays, suggesting that PL may be more effective in resolving DKA.

On the other hand, Gurnurkar et al. [10] discussed a case of an adolescent with newly diagnosed diabetes mellitus who developed severe hypercalcemia during DKA. The study emphasizes the importance of monitoring calcium levels during managing DKA in adolescents, as severe hypercalcemia is a rare and potentially life-threatening complication associated with DKA.

Jayashree et al. [3] conducted a double-blind, randomized controlled trial in a pediatric hospital in India, comparing the effectiveness of PL and NS as initial fluid therapies for children with DKA. The primary outcome was the frequency of new or progressive acute kidney injury (AKI), and secondary outcomes included DKA resolution time, changes in electrolyte levels, mortality rates, and lengths of ICU and hospital stays. The study found no significant differences in the outcomes between the two groups.

Gurnurkar et al. [10] highlighted a case study where a pediatric patient's severe hypercalcemia, a rare and life-threatening condition, was linked to DKA. They postulated that dehydration and metabolic acidosis could contribute to this condition and emphasized the importance of appropriate fluid selection, such as Plasma-Lyte A, in managing such cases effectively.

Oliver et al. [11] conducted a nested cohort study examining if the administration of PL in emergency departments could reduce the need for ICU admission for DKA patients compared to NS. Encompassing 84 patients, the study found no significant difference in ICU admission rates between the two groups.

In addition, Mahler [12] undertook a randomized, double-blind study involving 45 patients, investigating whether administering a balanced electrolyte solution (BES) could prevent hyperchloremic metabolic acidosis in patients with DKA. Patients treated with BES displayed a significant decrease in serum chloride levels and increased bicarbonate levels compared to those treated with NS, indicating the prevention of hyperchloremic metabolic acidosis.

In a secondary analysis of two cluster-randomized clinical trials involving 172 adults with diabetic ketoacidosis (DKA), the use of balanced crystalloids (such as Ringer's lactate or Plasma-Lyte A) was compared to standard saline (0.9% sodium chloride) for acute treatment. The study found that balanced crystalloids led to a faster resolution of DKA, with a median time of 13.0 hours, compared to 16.9 hours with saline. Additionally, the time to discontinue continuous insulin infusion was shorter in the balanced crystalloids group. These findings suggest that balanced crystalloids may be a better alternative to saline for the acute management of adults with DKA [13].

In pediatrics, a randomized controlled trial studied the efficacy of PL compared to 0.9% NS in treating patients with DKA to determine their impact on AKI [14]. The trial involved 66 children and assessed the incidence of new or progressive AKI and the DKA resolution time. The results revealed no significant difference between PL and 0.9% NS regarding the incidence of AKI, time to resolution of DKA, need for renal replacement therapy, mortality, or length of ICU and hospital stay. This indicates that both fluids are similarly effective in managing pediatric DKA. Another trial by the same team revealed 

Table 1 presents a succinct summary of the literature on treating DKA in adult and pediatric populations. This compilation is an invaluable resource for healthcare professionals, researchers, and academicians, as it brings together key findings and insights from various studies.

**Table 1:** Summary of the available literature.

Originating information			Framing of the research		Scope of the research		Research outcomes
Author/s	Year of Publication	Country of origin	Purpose/Aims	Epistemology / theoretical framework/ approach	Study population/ sample size	Limitations	Results
Ramanan et al. [7]	2021	Germany	To evaluate if PL resolves DKA faster than NS and if its acetate potentiates ketosis.	Cluster-crossover-randomized-controlled Phase 2 trial	Ninety-three patients were enrolled, with 90 in the modified-intention-to-treat population (PL = 48, NS= 42).	Small Sample size	At 48 h, the PL and NS groups had median blood ketones of 0.3 mmol/L (IQR 0.1–0.5) and a median anion gap of 6 mEq/L (IQR 5-7). DKA resolution at 48 h was 96% (PL) and 86% (SC); hazard ratio 3.93 (95% CI 0.73-21.16, p = 0.111). DKA resolution was 69% (PL) and 36% (SC) at 24 h; odds ratio 4.24 (95% CI 1.68–10.72, p = 0.002). PL and SC groups had median ICU and hospital stays of 49 h (IQR 23–72) vs. 55 h (IQR 41–80) and 81 h (IQR 58–137) vs. 98 h (IQR 65–195), respectively.
Chua et al. [8]	2012	Australia	The purpose of the study was to determine the effects of Plasma-Lyte 148 (PL) vs 0.9% saline (NS) fluid resuscitation in diabetic ketoacidosis (DKA).	A multicenter retrospective examination of people referred to the intensive care unit for DKA who received mostly solely PL or NS infusions for 12 hours was conducted.	55 eligible moderate-to-severe DKA patients were admitted during the research. 23 patients met the inclusion and exclusion criteria and were studied: 9 PL and 14 NS individuals.	Small Sample size	PL-resuscitated DKA patients experienced faster metabolic acidosis resolution, reduced hyperchloremia, and improved blood pressure and urine output.
Attokaran et al. [9]	2023	Australia	To test the hypothesis that fluid resuscitation in the ED with playmate-148 (PL) compared with 0.9% sodium chloride (NS) would result in a lower proportion of patients with diabetic ketoacidosis (DKA) requiring intensive care unit (ICU) admission.	Prespecified nested cohort study at two hospitals within a cluster, crossover, open-label, randomized, controlled trial	84 patients enrolled in the study, 38 (45%) in the SC group and 46 (55%) in the PL group	The poor compliance with allocated study fluid is a limitation that limits the generalizability of results.	The present study did not show a significant reduction in ICU admission rate or hospital LOS for patients presenting with DKA when treated with PL compared with SC as fluid therapy.
Oliver et al. [11]	2018	USA	This study evaluated plasma-lyte A (PL) and sodium chloride 0.9% (NS) for DKA resolution with one fluid, mostly for resuscitation.	A retrospective cohort Study.	84 patients. PL: 23; NS: 61.	Retrospective medical record review complicates causality and association. The study's small sample size may limit generalizability. Clinical use of NS and balanced electrolytes complicates our results.	Balanced electrolyte resuscitation or NS for DKA resolution. Not significant. Infusion duration, cumulative insulin dose at 24 and 48 hours, and hospital stay were unaffected.
Self et al. [13]	2020	USA	To compare saline and balanced crystalloids for acute DKA treatment in humans.	pragmatic, multiple-crossover, cluster-randomized clinical trials	This secondary cluster trial study included 172 adults, 94 of whom received PL and 78 NS.	This study was a subgroup analysis of prior clinical trials.	Analysis showed that the PL group had a shorter time to DKA resolution (median 13.0 hours; IQR: 9.5–18.8 hours) and a shorter time to insulin infusion discontinuation (9.8 hours; IQR: 5.1–17.0 hours) than the NS group (13.4 hours; IQR: 11.0–17.9 hours).
Williams et al. [14]	2020	India	Pediatric DKA with severe metabolic decompensation and high risk for AKI comparing Plasma-Lyte A (PL)with 0.9% saline(NS) as the first fluid.	the prospective, double-blind, parallel-assignment, investigator-initiated randomized controlled experiment	Estimated 60 participants (almost 30 in each group) were expected to meet one or more composite AKI criteria.	Despite being a composite variable, the primary outcome occurred rarely in both groups, undermining the study. Multicentric studies are necessary to increase the sample size in single-center studies.	AKI was common in children with DKA in this exploratory trial comparing NS versus PL as the first fluid. Both groups had similar AKI rates and resolutions. PL reduced AKI, DKA, RRT, mortality, and PICU/hospital stay similarly to NS.

## Conclusions

Plasma-Lyte (PL) has been shown to enhance the treatment of Diabetic Ketoacidosis (DKA) compared to Normal Saline (NS). Patients receiving PL experienced a rise in bicarbonate levels, blood pressure, and reduced chloride and potassium levels. Additionally, metabolic acidosis was found to be reversed more swiftly with PL. Evidence suggests that using PL may expedite the resolution of DKA without worsening ketosis and may also contribute to shortened ICU and hospital stays. In contrast, when comparing the incidence of Acute Kidney Injury (AKI), mortality rates, and lengths of hospitalization, PL and NS did not exhibit significant differences. This indicates that both fluids are relatively comparable in managing these specific aspects of DKA. Remarkably, Plasma-Lyte A has been identified as an effective fluid in managing pediatric cases of DKA accompanied by hypercalcemia. This has been attributed to its balanced electrolyte composition, instrumental in rectifying metabolic imbalances. In emergency departments, both PL and NS demonstrated similar rates of ICU admissions for DKA patients, suggesting that the choice between these fluids may not significantly impact the necessity for intensive care. For adult patients with DKA, PL has proven superior in mitigating hyperchloremia and accelerating the resolution of metabolic acidosis compared to NS. The conclusion drawn here relies on the data collected during this review. To reinforce these findings, we must conduct additional Randomized Controlled Trials (RCTs) and a comprehensive systematic review and meta-analysis.
